# *BNDF* methylation in mothers and newborns is associated with maternal exposure to war trauma

**DOI:** 10.1186/s13148-017-0367-x

**Published:** 2017-06-30

**Authors:** Darlene A. Kertes, Samarth S. Bhatt, Hayley S. Kamin, David A. Hughes, Nicole C. Rodney, Connie J. Mulligan

**Affiliations:** 1 Department of Psychology and University of Florida Genetics Institute, 945 Center Drive, Gainesville, FL 32611-2250 USA; 2 Department of Psychology, University of Florida, Gainesville, FL USA; 3 Integrative Epidemiology Unit, University of Bristol, Bristol, UK; 4 Department of Anthropology, University of Florida, Gainesville, FL USA; 5 Department of Anthropology and University of Florida Genetics Institute, University of Florida, Gainesville, FL USA

**Keywords:** Brain-derived neurotrophic factor, *BDNF*, Stress, Trauma, War, Prenatal, DNA methylation, Transcription factor, Blood, Placenta

## Abstract

**Background:**

The *BDNF* gene codes for brain-derived neurotrophic factor, a growth factor involved in neural development, cell differentiation, and synaptic plasticity. Present in both the brain and periphery, BDNF plays critical roles throughout the body and is essential for placental and fetal development. Rodent studies show that early life stress, including prenatal stress, broadly alters *BDNF* methylation, with presumed changes in gene expression. No studies have assessed prenatal exposure to maternal traumatic stress and *BDNF* methylation in humans. This study examined associations of prenatal exposure to maternal stress and *BDNF* methylation at CpG sites across the *BDNF* gene.

**Results:**

Among 24 mothers and newborns in the eastern Democratic Republic of Congo, a region with extreme conflict and violence to women, maternal experiences of war trauma and chronic stress were associated with *BDNF* methylation in umbilical cord blood, placental tissue, and maternal venous blood. Associations of maternal stress and *BDNF* methylation showed high tissue specificity. The majority of significant associations were observed in putative transcription factor binding regions.

**Conclusions:**

This is the first study in humans to examine *BDNF* methylation in relation to prenatal exposure to maternal stress in three tissues simultaneously and the first in any mammalian species to report associations of prenatal stress and *BDNF* methylation in placental tissue. The findings add to the growing body of evidence highlighting the importance of considering epigenetic effects when examining the impacts of trauma and stress, not only for adults but also for offspring exposed via effects transmitted before birth.

**Electronic supplementary material:**

The online version of this article (doi:10.1186/s13148-017-0367-x) contains supplementary material, which is available to authorized users.

## Background

Throughout life, brain-derived neurotrophic factor (BDNF) acts as a key regulator of neuronal development and activity including axonal growth, maturation and survival of neurons, and synaptic plasticity [1]. This neuronal growth factor, encoded by the gene *BDNF*, is a member of the neurotrophin family of polypeptide growth factors that are widely expressed throughout the brain and impact a broad range of brain functions [2, 3]. BDNF crosses the blood-brain barrier and is present in the bloodstream and peripheral tissues as well, exerting neuro-protective effects throughout the body [4–6]. During the prenatal period, BDNF potentiates placental development and facilitates cytotrophoblast differentiation, proliferation, migration, and survival necessary for fetal growth [7, 8].

The structure and sequence of the *BDNF* gene is complex but highly conserved across species [9]. In humans, it contains 11 exons, nine of which contain promoters that regulate its expression [10]. Regulation of BDNF is also tightly controlled by multiple transcription factors and by intracellular signaling pathways that involve binding to specific receptors [11]. To add to the complexity, exons and transcripts vary in their expression across neural structures and other tissues, and across development both pre- and postnatally [12].

BDNF has widespread functional consequences for development. Rodent studies implicate *BDNF* expression in learning and memory, aggressiveness, anxiety-like behavior, and fear memory formation and consolidation [13–16]. In humans, BDNF levels are altered in attentional, neurodevelopmental, and stress-related mood and anxiety disorders [17–21]. At birth, higher maternal blood but lower cord blood and placental BDNF levels are associated with prematurity and lower gestational age [22–26], with cord blood levels also predicting birth complications [27, 28].

Bidirectional influences between BDNF levels and the stress-sensitive hypothalamic-pituitary-adrenocortical (HPA) axis implicate a role for stress in BDNF signaling. Elevated levels of glucocorticoids, released from the HPA axis following stress, interfere with BDNF signaling [29]; at the same time, BDNF and glucocorticoids co-regulate activation of the HPA axis [30].

Robust evidence for stress-induced changes in behavior and BDNF levels in several brain regions comes from animal models [31–33] of experimental stress administered to mothers before or during pregnancy and in the early postnatal period [34–37]. Animal models also show that early life stress exposure induces changes in *BDNF* methylation, though the direction of effects varied by developmental timing of exposure, age at testing, tissue source, and *BDNF* loci tested [35, 38–42]. Stress-induced epigenetic changes at the exon IV promotor are the most widely reported, although effects have been observed across the *BDNF* gene. Interestingly, stress-induced epigenetic modifications to *BDNF* can be transmitted intergenerationally and are not reversed by cross-fostering, suggesting they occur prenatally during exposure to the intrauterine environment [43].

Less is known about the effects of stress exposure on *BDNF* methylation in humans. Among adults, *BDNF* methylation levels in blood or saliva are associated with anxiety, major depressive disorder, post-traumatic stress disorder, and a history of childhood maltreatment or exposure to domestic violence [44–49]. In two studies of psychiatric patients, a history of childhood maltreatment was associated with methylation in sites homologous to genomic regions identified in rodent studies in exons 1 and 4 [44, 45]. In exon 4, associations were observed almost exclusively at putative transcription factor binding (TFB) sites with important regulatory control over gene expression [45].

Only two studies have investigated *BDNF* methylation in relation to prenatal emotional state, both of which examined maternal depressive symptoms with methylation levels quantified at the exon 4 promotor. In one study, no relation of depressed mood to *BDNF* methylation was observed in neonatal cord blood [50]. In the other, exposure to prenatal depressive symptoms significantly predicted decreased methylation of *BDNF* in buccal cells at 2 months of age [51].

To date, there are no published reports examining prenatal exposure to maternal traumatic stress and *BDNF* gene methylation in humans. Moreover, despite knowledge that BDNF plays a key role in regulating placental and fetal development [7, 52], there are no reports, either human or animal, testing prenatal stress exposures and *BDNF* methylation in placenta. The present study addresses these gaps by examining prenatal exposure to maternal psychosocial stress among mothers and newborns in a war-torn region of the eastern Democratic Republic of Congo. The Congo has experienced military and civilian conflict for more than two decades and is routinely described as the site of one of the world’s worst humanitarian crises [53]. Sexual violence and other acts of terror, aggression, and human rights violations are widespread and often co-occur with chronic social inequity and economic strain [54–56]. We have previously documented in this population associations of war trauma and chronic stress with DNA methylation in genes regulating the HPA axis [57, 58].

Here, we report findings suggesting that maternal experiences of war trauma and chronic stress are related to DNA methylation levels across the *BDNF* gene in maternal blood, umbilical cord blood, and placental tissue. In placenta, we also conducted targeted sequencing of the *BDNF* exon 4 promotor, based on prior research in rodent brain and human blood samples [11, 35], to determine whether stress-linked epigenetic alterations could be detected. We predicted that prenatal exposure to maternal stress would be associated with *BDNF* methylation in both newborn and maternal tissues. Because of the complexity of the *BDNF* gene and evidence from animal models that the direction of effects vary by tissue source and gene locus [41, 59], directional hypotheses regarding hypo- or hypermethylation in each tissue were not posited. Based on our findings with genes regulating the HPA axis [57], we expected war traumas, due to their severity, would be a stronger predictor of DNA methylation than chronic stress. Based on the high degree of conservation across species of *BDNF* regions functionally relevant for gene transcription [9], we highlight stress—methylation associations for CpG sites situated at putative transcription factor binding regions.

## Results

**Fig. 1:**
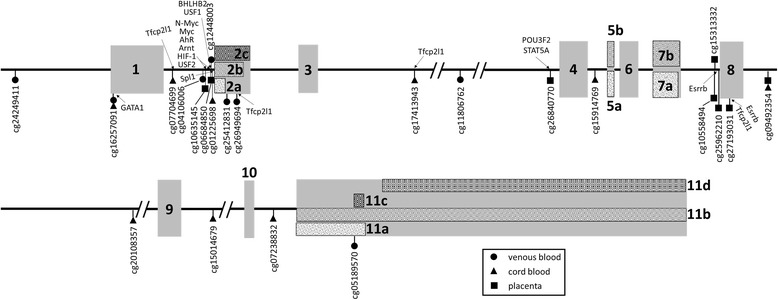
Schematic representation of human *BDNF* gene, depicted from the NCBI reference number NG_011794.1 [103]. Significant CpG sites are shown relative to intronic/exonic regions, HMR conserved TFBs, and putative TFB sites identified in Physbinder and MotifMap. Gray boxes represent exons. Exons on different splice variants are indicated by lowercase letters and the exon numbers proposed by Pruunsild et al. [10] are in roman numerals

We examined methylation levels at 67 CpG sites across the *BDNF* gene via the HumanMethylation450 BeadChip in maternal venous blood, umbilical cord blood, and placental tissues. Analyses were conducted using beta regression such that positive regression coefficients reflected higher methylation associated with higher stress, whereas a negative regression coefficient was indicative of lower methylation associated with higher stress. The results showed that a total of 23 CpG sites were significantly associated with chronic stress or war trauma in one or more tissues at *p* < .05 (Table 1). A total of 20 sites were associated with war trauma and nine with chronic stress. Of those, six sites were predicted by both stressors (Table 1, italicized sites). Twelve sites survived false discovery rate (FDR) correction with moderate confidence (*q* < .25); 11 with war trauma and one with both war trauma and chronic stress. For nominally significant sites (*p* < .05), findings were considered meaningful and thus described here if at least one of the following two a priori criteria were met: previous association of the CpG with traumatic stress or brain and behavioral functioning, or situated at known or putative TFBs [57] (indicated in Table 1). The results indicated that all of the CpG sites with a prior history of association in human or animal studies were situated at TFBs. Locations of CpG sites situated at or near TFBs are shown in Fig. 1. TFB region information is available in Additional file 1: Table S1.

**Table 1:** Association estimates of stress exposures and methylation of CpG sites using beta regression

CpG	Tissue	Position	Gene region^a^	Upstream of exon	Putative TFB^b^	Regres coeff^c^	*p*	*q*	*R* ^*2*^	Abs beta	∆ beta
War trauma											
**cg24249411**	**Venous**	**27744760**	**Intergenic**	**1**	**CTCF** ^**e**^	**–3.86**	**.02**	**.09**	**.22**	**.14**	**.07**
**cg16257091** ^1^	**Venous**	**27743580**	**Core promoter**	**2**	**GATA1** ^**f**^	**–3.07**	**.05**	**.15**	**.17**	**.14**	**.15**
cg16257091 ^1^	Placenta	27743580	Core promoter	2	GATA1^f^	2.08	.02	.27	.18	.29	.51
*cg16257091* ^1^	Cord	27743580	Core promoter	2	GATA1^f^	4.82	.02	.27	.21	.19	.10
cg07704699	Cord	27742833	Intragenic	2	Tfcp2l1^f^	**–**2.93	.01	.27	.25	.22	.19
***cg04106006***	**Venous**	**27742455**	**Proximal promoter/intragenic**	**2**	**n/a**	**–1.92**	**.03**	**.11**	**.19**	**.36**	**.23**
cg10635145	Placenta	27742436	Proximal promoter/intragenic	2	n/a	1.93	.03	.28	.19	.71	.30
***cg06684850***	**Venous**	**27742369**	**Proximal promoter/intragenic**	**2**	**N-Myc** ^**d, f**^ **, Myc** ^**d**^ **, AhR** ^**d**^ **, Arnt** ^**d**^ **, HIF-1** ^**d**^ **, USF2** ^**f**^	**–3.73**	**.05**	**.15**	**.16**	**.11**	**.12**
**cg12448003**	**Venous**	**27742366**	**Proximal promoter/intragenic**	**2**	**N-Myc** ^**d,f**^ **, Myc** ^**d**^ **, AhR** ^**d**^ **, Arnt** ^**d**^ **, HIF-1** ^**d**^ **, USF1** ^**d**^ **, USF2** ^**d,f**^ **, BHLHB2** ^**f**^	**–18.16**	**.01**	**.09**	**.26**	**.03**	**.02**
**cg25412831**	**Venous**	**27742138**	**Intragenic**	**3**	**n/a**	**–5.61**	**.03**	**.17**	**.18**	**.09**	**.10**
***cg26949694***	**Venous**	**27742061**	**Intragenic**	**3**	**CTCF** ^**e**^ **, Tfcp2l1** ^**f**^	**–3.13**	**.02**	**.09**	**.23**	**.18**	**.16**
**cg17413943**	**Cord**	**27739827**	**Intragenic**	**4**	**Tfcp2l1** ^**f**^	**–8.15**	**.003**	**.18**	**.33**	**.08**	**.05**
**cg11806762**	**Venous**	**27732958**	**Intragenic**	**4**	**n/a**	**7.46**	**.002**	**.05**	**.34**	**.91**	**.10**
cg26840770^2^	Placenta	27723291	Proximal promoter/intragenic	4	CTCF^e^, Pol2^e^, POU3F2^g^, STAT5A^g^	**–**11.97	.03	.28	.21	.04	.04
cg15914769	Cord	27722775	Intragenic	5	n/a	17.97	.03	.28	.17	.03	.02
**cg10558494**	**Placenta**	**27721280**	**Proximal promoter/intragenic**	**8**	**n/a**	**5.27**	**.01**	**.12**	**.17**	**.09**	**.25**
**cg15313332**	**Placenta**	**27721270**	**Proximal promoter/intragenic**	**8**	**n/a**	**7.14**	**.002**	**.06**	**.22**	**.07**	**.21**
***cg25962210***	**Placenta**	**27721223**	**Core promoter/intragenic**	**8**	**Esrrb** ^**f**^	**9.61**	**.001**	**.06**	**.30**	**.06**	**.10**
cg27193031	Placenta	27721088	Intragenic	9	Esrrb^f^, Tfcp2l1^f^	4.48	.05	.38	.12	.09	.16
*cg09492354*	Placenta	27720710	Intragenic	9	CTCF^e^, Pol2-4H8^e^	**–**11.32	.02	.27	.20	.04	.02
*cg09492354*	Cord	27720710	Intragenic	9	CTCF^e^, Pol2-4H8^e^	9.46	.03	.28	.19	.05	.02
cg20108357	Cord	27718979	Intragenic	9	n/a	1.74	.05	.42	.15	.33	.21
**cg05189570**	**Venous**	**27680481**	Intragenic	**11**	**n/a**	**4.75**	**.02**	**.09**	**.23**	**.89**	**.10**
Chronic stress											
***cg16257091*** ^1^	**Cord**	**27743580**	**Core promoter**	**2**	**GATA1** ^**f**^	**5.80**	**.01**	**.19**	**.26**	**.11**	**.10**
cg01225698^3^	Cord	27742355	Proximal promoter/intragenic	2	Spl1^f^	6.70	.05	.32	.15	.06	.09
*cg04106006*	Venous	27742454	Proximal promoter/intragenic	2	n/a	**–**1.88	.03	.85	.19	.39	.23
*cg06684850*	Venous	27742369	Proximal promoter/intragenic	2	N-Myc^d,f^, Myc^d^, AhR^d^, Arnt^d^ **,** HIF-1^d^, USF2^f^	**–**4.28	.02	.85	.21	.11	.12
*cg26949694*	Placenta	27742061	Intragenic	3	CTCF^e^, Tfcp2l1^f^	1.70	.05	.42	.15	.34	.54
*cg25962210*	Placenta	27721223	Core promoter/intragenic	8	Esrrb^f^	8.15	.02	.42	.20	.06	.10
*cg09492354*	Placenta	27720710	Intragenic	9	CTCF^e^, Pol2-4H8^e^	**–**10.78	.04	.42	.17	.04	.02
*cg09492354*	Cord	27720710	Intragenic	9	CTCF^e^, Pol2-4H8^e^	9.34	.05	.32	.15	.05	.02
cg15014679	Cord	27695210	Intragenic	10	n/a	5.56	.05	.32	.13	.91	.13
cg07238832	Cord	27681475	Intragenic	11	n/a	4.33	.04	.32	.17	.89	.13

*Note*: Underlined CpGs are significant across tissue. Italicized CpGs are significant across stressor type. Bolded lines have *q* value <.25. Due to potential error in array data at the extremes of the beta distribution, CpG sites with absolute (mean) methylation <5% or >95% should be interpreted with caution. *R* ^2^ values indicate percent variance explained (after controlling for infant sex in cord blood and placenta)

*n/a* not available

^a^Multiple regions are indicated if differing according to *BDNF* transcript

^b^Putative TFBs were identified for purposes of this study via in silico modeling or molecular assay not specific to tissue source

^c^Regres coeff refers to the regression coefficient from beta regressions

^d^TFB identified via MotifMap

^e^TFB identified via Encode

^f^TFB identified via PhysBinder (avg precision)

^g^TFB identified via HMR Conservation

^1^Smith et al. [47]

^2^Thaler et al. [45]; Fuchikami et al. [77]; Martinowich et al. [76]; Dennis et al. [75]

^3^Weder et al. [46]

War trauma predicted methylation at 16 sites, explaining 12–34% of the variance as indicated by *R* ^*2*^ values (Table 1). Chronic stress predicted methylation at six sites, explaining 13–26% of the variance. Significant associations were observed in all three tissue types for both stressors. Methylation correlations among each pair of tissues were computed; none of the CpG sites in Table 1 had significant cross-tissue correlations.

Methylation levels at nine CpG sites were associated with war trauma in maternal blood: cg2429411, cg16257091, cg04106006, cg06684850, cg12448003, cg25412831, cg26949694, cg118067262, and cg05189570, all of which survived FDR correction at moderate confidence (*q* range = .05–.17; Table 1). Of the nine significant sites, five were also located at putative binding regions of one or more transcription factors: cg2429411, cg16257091, cg06684850, cg12448003, and cg26949694, with cg16257091 also previously identified in the literature (TFBs and citations shown in Table 1). Methylation at cg04106006 and cg06684850 was also nominally associated with chronic stress.

In placenta, methylation at cg10558494, cg15313332, and cg25962210 was significantly associated with war trauma after FDR correction (Table 1). cg25962210 was located at a TFB and also nominally associated with chronic stress. Several additional sites did not survive FDR correction but were all situated within one or more TFBs. These were cg16257091, cg28640770, and cg27193031, associated with war trauma, cg26949694, associated with chronic stress, and cg09492354, associated with both stressor types. cg26840770 has been previously identified in animal models and human studies (see Table 1).

In cord blood, methylation at two CpG sites was significantly associated with stressors after FDR correction: cg17413943 with war trauma and cg16257091 with chronic stress. The latter was the only CpG site associated with chronic stress at FDR-corrected significance. Three additional CpG sites were nominally significant but located at multiple putative TFBs: cg01225698, cg09492354, and cg07704699, with cg01225698 also showing prior association with traumatic stress.

Most significant associations were tissue specific. There were three CpG sites significant in more than one tissue (cg16257091, cg26949694, and cg09492354). In all cases, the direction of effect differed across tissues. At cg16257091, higher maternal stress was associated with lower methylation in maternal blood but higher methylation in placenta and cord blood. Similarly, higher maternal stress was associated with lower methylation at cg26949694 in maternal blood but higher methylation in placenta. Opposing directions were also observed at a third CpG site, cg09492354; however, the low absolute level of methylation at that site (4–5%) warrants caution in its interpretation.

Methylation at several CpG sites (cord blood cg16257091 and cg09492354; maternal blood cg04106006 and cg06684850; placenta cg25962210 and cg09492354) was associated with both stressor types in the same tissue. Post hoc analyses comparing a one-stressor predictor model with a two-stressor predictor model revealed no improvement in model fit with the two-stressor model (data not shown). This indicated that associations observed across stressors were due to shared variance among the two stressor types.

For the sodium bisulfite sequencing data specifically targeting the exon 4 promotor in placental tissue, an initial set of 9 of the 24 placentas were randomly selected for sequencing. Across an average of 20 clones (range 18–22) per CpG site, methylation levels were uniformly low in placental tissue (*M* = .003, SD = .003). An additional 10 samples were sequenced with no substantial change in overall mean methylation levels (*M* = .003, SD = .004). Of the 19 CpG sites sequenced, nine showed any degree of variation, and the remaining 11 sites were uniformly unmethylated in all clones (see Additional file 1: Table S2). Targeted bisulfite sequencing was discontinued for the remaining five samples as it was deemed unlikely that meaningful statistical analyses could be computed with the limited variability that was observed in methylation levels at this region.

## Discussion

This study reports for the first time a significant association of prenatal maternal traumatic stress exposure with *BDNF* methylation in humans. Although only 47% of the total CpG sites assayed were situated near putative TFBs, a majority (66%) of the significant sites were in putative TFB regions. This finding is consistent with another study that similarly reported an over-representation of significant associations at TFB sites when examining links between *BDNF* methylation and history of childhood maltreatment among adult psychiatric patients [45].

Previously, we reported that war traumas had stronger associations than chronic stress with methylation of genes regulating the HPA axis [57]. Here, we add to the literature on stress and epigenetics by showing that more severe war traumas also are more strongly related than chronic stress to methylation in a key gene regulating neuronal and placental development. This was evident in that all but one of the associations that survived multiple test correction at moderate confidence using FDR-adjusted *q* values were with war trauma. Stress associations with the HPA genes and *BDNF* differed, however, in one potentially interesting way. For the HPA genes, unique, additive effects of chronic stress and war trauma were observed when both stressors were included in a single statistical model for those sites at which methylation was associated with both types of stress. In contrast, comparison of a one-stressor to a two-stressor model for *BDNF* methylation indicated that chronic stress did not add additional explanatory value above and beyond the stronger associations observed for war trauma for the sites at which methylation was associated with both stressors.

The observation that war traumas rather than chronic stress carried the bulk of the significant effects is noteworthy. A hallmark principle in research on the biology of stress is that biological effects are more likely to be detected for stressors that have a high degree of uncontrollability and unpredictability [60]. The war trauma index assessed in this study included such experiences as being kidnapped, raped, having family members killed, and being a refugee, which are arguably more severe, unpredictable, and uncontrollable than the chronic socioeconomic and socioemotional stressors captured by the chronic stress index. Indeed, other studies of more mild prenatal stress have not shown notable associations with offspring methylation, at least when tested in cord blood [61]. Despite detecting some links with chronic stress, the preponderance of findings suggest broader impacts of more severe war traumas on DNA methylation.

Extensive evidence points to tissue specificity in DNA methylation patterns (e.g., [62–65]). However, *BDNF* methylation has revealed an especially complex pattern of influences. In rodents, the same experimental stressor may differentially impact *BDNF* methylation, in terms of the significance and direction of effect, depending on whether the stressor occurs early in life vs. adulthood, the age at testing, the specific tissue source, and the *BDNF* loci targeted [38, 40, 41, 59]. Such complexity is also expected in peripheral tissues and in the prenatal period, as BDNF serves multiple critical roles for placental and fetal development that differ from its roles in the mature brain. Here, we document the links of extreme stress with methylation in three tissues collected at the time of birth: maternal blood, placental tissue, and umbilical cord blood. The results showed high tissue specificity. This is in line with the life cycle model of stress positing that stress effects differ by phase of the life course [66], developmental models of health and disease proposing high fetal sensitivity to intrauterine environmental cues [67], and tissue and developmental specificity of mammalian DNA methylation patterns.

In maternal blood, most of the significant CpG sites were located upstream of exons 1 through 4, where multiple splicing sites produce *BDNF* isoforms. In the intergenic region upstream of exon 1, cg24249411 was associated with war trauma after FDR correction. This CpG site was situated near a putative peak point position of CTCF binding, which regulates chromatin structure and defines active DNA boundaries to promote or repress gene transcription [68]. In the core promotor of exon 2, cg12257091 was significantly associated with war trauma at FDR-corrected significance. This CpG was located at the putative binding site for GATA1, a transcriptional activator or repressor believed to promote erythroid development. Methylation at this site in adult peripheral blood has previously been identified as associated with post-traumatic stress disorder [47]. Three additional CpG sites (cg04106006, cg0668450, and cg12448003) located in the proximal promotor or intragenic region upstream of exon 2 were significantly associated with maternal stress at FDR-corrected significance levels.

Observed associations of maternal stress with methylation at cg0668450 and cg12448003 are theoretically compelling because they are situated at multiple TFBs. cg0668450 was located in a region containing putative sites for six transcription factors, including N-Myc, Myc, AhR, Arnt, HIF-1, USF2, and cg12448003 is at putative binding sites for eight TFBs, including N-Myc, Myc, AhR, Arnt, HIF-1, USF1, USF2, and BHLHB2. Several of these transcription factors have known effects on *BDNF* expression or neural function, specifically, BHLHB2, USF2, and MYCN (Fig. 1 and Additional file 1: Table S1). BHLHB2 is known to control *BDNF* promoter activity and neuronal excitability [69], and USF2 acts as a transcriptional activator at a number of activity-regulated promoters [70]. MYCN is highly expressed in brain and critical for normal brain development [71]. Recently, it has been found that MYCN co-localizes and interacts with methyl-CpG-binding protein 2 (MeCP2) [72]. Of note, cg12448003 is within a “TCACGTGC” sequence. This is a cyclic adenosine monophosphate (cAMP)-responsive element (CRE)-binding protein response element “TGACGTCA”-like sequence with base substitutions at −2, −7, and −8 positions. This is potentially meaningful because it has already been observed that CRE-like elements, with base substitutions, serve as CREB binding sites [73] and binding of cAMP at a CRE plays a central role in neural activity-dependent *BDNF* gene expression by enhancing transcriptional activity [9, 11]. In addition, cg26949694, associated with war trauma in maternal blood, was located in the intragenic region upstream of exon 3 at the putative binding site for Tfcp2l1, which suppresses DNA transcription, and near the peak point of a putative binding region for CTCF. Collectively, the presence of a binding site for cAMP as well as multiple transcription factors, including those known to specifically affect *BDNF* gene expression or neural function, suggests potential functional consequences of war trauma on *BDNF*. Even for those CpG sites at which putative TFBs were identified, the functional role of transcription factors at the putative binding regions on gene activity and DNA methylation in these tissues requires experimental validation and further investigation.

Methylation at three additional CpG sites was significantly associated in maternal blood with war trauma at FDR-corrected significance: cg25412831, cg11806762, and cg05189570. As these CpG sites were not at documented TFBs, the functional relevance of these findings is unknown.

With respect to findings in placental tissue, war trauma was associated after FDR correction with methylation at three CpG sites upstream of exon 8. cg25962210, associated with both war trauma and chronic stress, is located in the core promotor or intragenic region of exon 8, depending on splice variant. This CpG site is situated at a putative binding site for Esrrb, which is involved in both stress regulation and placental development [74]. Also significantly associated with war trauma were cg10558494 and cg15313332, both situated at the proximal promotor (or intragenic) region of exon 8 with no known TFBs. Maternal stressors were nominally associated with additional CpG sites in the intragenic region upstream of exons 3, 4, and 9. They included cg26949694, situated near the peak point position of a putative CTCF binding region and at the putative binding site of Tfcp2l1, cg27193031, situated at Esrrb and Tfcp2l1 putative binding sites, and cg09492354, situated near the peak point positions of the putative binding regions for CTCF and Pol2-4H8, the largest subunit of polymerase II. Also significant in placental tissue was cg26840770, which was situated near the peak points of the CTCF and POL2 putative binding regions and at the HMR conserved binding sites for POU3F2 and STAT5A. POU3F2 is involved in neuronal differentiation and enhances the activation of corticotrophin-releasing hormone regulated genes. STAT5A is a transcriptional activator that mediates the responses of many cell ligands. cg26840770 methylation has been previously identified in studies of brain function in rodents [75, 76] and stress-related mental disorders in humans [45, 77].

In cord blood, maternal stress predicted methylation at five sites located in TFB regions, the majority of them upstream of exon 2. Among them was cg16257091, located in the core promotor of exon 2, which was nominally associated with war trauma and significantly associated after FDR correction with chronic stress. This CpG site was situated at the putative binding site of GATA1, which regulates the switch of fetal hemoglobin to adult hemoglobin. Also upstream of exon 2, cg07704699 methylation was associated with war trauma. This CpG site was situated at the putative binding sites for Tfcp2l1. cg01225698, located in the proximal promotor or the intragenic region depending on splice variant, was situated at a putative binding site for Spl1, which reduces DNA binding. Methylation at this site has previously been associated with child maltreatment [46]. Another noteworthy finding in cord blood was the significant association after FDR correction of war trauma with cg17413943, located upstream of exon 4 in the intragenic region and at a Tfcp2l1 putative binding site. cg09492354, located in the intragenic region upstream of exon 9, was nominally associated with war trauma and chronic stress. Although caution is warranted in interpreting cg09492354 given the very low absolute methylation level, this CpG is in a genomic region near the peak point position of the putative binding regions for CTCF and Pol2-4H8.

The sequencing data of the exon 4 promotor region revealed that the region was almost entirely unmethylated in placental tissue. Although the low methylation levels precluded analyzing DNA methylation variation in relation to maternal stress, the low levels in themselves are noteworthy. The region sequenced encompasses a transcription start site as well as a cAMP response element. Moreover, this exon contains binding sites for MeCP2, known to suppress gene transcription, and CREB, an upstream transcriptional activator of BDNF [11, 78]. It is believed that cAMP binding plays a central role in neural activity-dependent *BDNF* gene expression by enhancing transcriptional activity [9, 11]. In animal models, prenatal stress exposure impacts methylation in this region among offspring in the amygdala and hippocampus [35]. Given the important role that BDNF plays in placental and fetal development, it is notable that this important regulatory region was nearly entirely unmethylated in placenta and therefore fully accessible to transcription factors. It also suggests that transcription factors that bind to this region may play an important role in BDNF regulation in placenta.

A limitation of this study is the relatively small sample size. We were, however, able to detect significant associations even with FDR correction, and our sample size is comparable to other recent human methylation studies (*N* = 19–36), including studies examining the effects of psychosocial stress [79–83]. The small sample size precluded testing potential associations of stress and methylation for newborn boys and girls separately. Some rodent studies examining *BDNF* methylation have observed sex-specific effects in brain (e.g., [37, 40]). However, a recent human study documenting an association of prenatal maternal depressive symptoms with *BDNF* methylation in both male and female infants suggests no sex difference, at least in human peripheral tissue [51].

## Conclusions

*BDNF* transcripts are heterogeneously expressed in brain regions and in the periphery, the complexity of which has yet to be fully understood [3, 10]. Because of this heterogeneity, we do not assume that *BDNF* methylation in peripheral tissues is the same as in a particular brain region. Nevertheless, examining *BDNF* methylation in these tissues is relevant for prenatal development as *BDNF* plays a role in placental and fetal growth [7] and BDNF protein levels in the periphery are associated with birth outcomes [23–25]. Recent research has documented several types of prenatal exposures, including air pollution and nicotine use, are associated with placental *BDNF* methylation [84, 85]. Our study is the first in humans to examine *BDNF* methylation patterns in relation to prenatal exposure to maternal stress in three tissues simultaneously and the first in any mammalian species to report prenatal stress—*BDNF* methylation associations in placental tissue.

The findings from this research add to the growing body of evidence highlighting the importance of considering epigenetic effects when examining the impacts of trauma and stress, not only for adults but also for offspring exposed via effects transmitted before birth. This knowledge highlights the potential intergenerational impact of traumatic stress to inform preventive intervention among vulnerable populations.

## Methods

### Participants

Participants were 24 mothers and their newborns living in the eastern Democratic Republic of Congo, a region with long-term military and civilian conflict and a history of severe violence against women. Participants were recruited from HEAL Africa hospital, Goma. Any mother who gave birth at the hospital during the period of data collection was eligible for inclusion. Participation was voluntary and confidential.

Informed consent was obtained under approval from Western IRB, the University of Goma, and an ethical review committee at HEAL Africa hospital, Goma. During oral consent, mothers were provided with a detailed explanation of the project and the possible uses of results and encouraged to ask questions about the study and its objectives.

Mean maternal age was 26.9 years (SD = 5.6), and 29% of mothers were primiparous (*N* children range = 1–9). The majority of women were married (79%) and co-habitating with the father of their child (75%). All mothers self-reported as non-smokers. The majority of births were vaginal deliveries (83%). Fifty-four percent of infants were male.

### Procedure

Umbilical cord blood, maternal venous blood, and placental tissue from the largest fetal cotyledon were obtained shortly after birth. A cord blood sample was not obtained from one newborn, yielding 23 cord blood, 24 placenta, and 24 maternal blood samples.

To obtain maternal report of stress exposure, mothers were interviewed within one day after birth regarding general health, reproductive history, chronic socioeconomic and socioemotional stress, and war-related traumas using culturally sensitive, semi-structured ethnographic interviews [86]. Interviews were conducted in the Congolese dialect of Swahili and followed an oral history format designed to facilitate rapport and emphasize open dialogue between the participant and interviewer. Questions assessed stressors relevant to this cultural setting, while also covering constructs measured in standardized stress and trauma inventories [87, 88].

Interviews were transcribed and coded for the presence or absence of 32 stressors. From this initial pool of items, two composite scales were generated that reflected Chronic Stress (19 items) and War Trauma (10 items). The construct and content of the stressor scales has previously been described in detail [57]. Briefly, chronic stress included items such as an unhappy marriage and difficulty paying bills. War traumas reflected experiences as a refugee, war-related rape, and exposure to armed conflict. The content of these two scales was determined using factor analysis. Two items (in-law stress and co-wives) that did not load with the other stress items were dropped, as was a third item pertaining to rape that showed redundancy with another question. Total scores were computed as an additive count of the number of chronic stress and war trauma items endorsed. There was wide variability in the presence and number of stressors reported. Fifty-five percent of mothers reported experiencing at least one war trauma (*M* = 1.58, SD = 2.21; range = 0–8). Of those, 31% reported one event, and an equal proportion of 23% reported two, three, or four or more events. The most commonly reported events were having been raped and/or a refugee as a result of war. Chronic stress also showed wide variability (*M* = 6.54, SD = 6.32; range = 0–18), with 33% reporting few (0–1) stressors and the remainder reporting higher levels of chronic stress. Internal consistency of the final scales was high with Cronbach’s α > .80 for each. As we have reported previously [57], the correlation between the scales was high (*r* = .74, *p* < .001).

### Epigenotyping

Genomic DNA was extracted from all tissues using Qiagen QIAamp DNA Midi Kits (Qiagen, Valencia, CA) and treated with sodium bisulfite. Samples (500 ng) were processed on Illumina HumanMethylation450 BeadChips at the University of Miami Hussman Institute for Human Genomics in Miami, FL, with output processed through Illumina’s GenomeStudio V2011.1 Methylation Module v1.9.0. The chip epigenotyping method enables assessment of CpG sites distributed broadly across the gene region. Methylation was indexed via average beta estimates. Quality control was evaluated using detection *p* value such that a value below the detection *p* value threshold indicated successful detection. In this study, any probe with a *p* value >.01 in any one sample was eliminated in all tissues. Using data from the 1000 Genomes Project, AFR population [89], we removed probes containing any of the following variation: (1) an observed variant, at any frequency, at the targeted CpG site, (2) an observed variant with an allele frequency ≥5% in the probe sequence, and (3) three or more variants at any frequency within the probe sequence. Using public data from Chen et al. (2011), probes that cross-hybridize with unspecific genomic targets were removed. After data filtering, type I and type II probes were normalized using the BMIQ package [90] as implemented in R [91]. Output from a total of 67 probes from across the *BDNF* gene were generated.

Several publicly available bioinformatics resources were used to identify putative TFBs. UCSC genome browser [92] was used to find HMR conserved TFBs and the proximity of putative ENCODE TFB regions to the CpG sites assayed via chip sequencing. The presence of putative TFBs flanking a given CpG site was further assessed using PhysBinder [93], with the threshold level set to average precision, and MotifMap [94]. See Additional file 1: Table S1 for the specific locations of putative transcription factor binding regions.

### Sodium bisulfite sequencing

A 376-bp region containing 19 CpG sites from the exon 4 promoter (Additional file 1: Figure ﻿S1) ﻿in placenta was amplified in two rounds of polymerase chain reaction. Fragments were amplified using HotStarTaq Polymerase (Qiagen, Valencia, CA) and the following primers: forward TTTTTGGTGTTYGGTATGTATTTTTTTTGTTTTGTAG and reverse AAAAACATACAATACCCTAAAAC. The PCR reaction (25 μl) was loaded on an agarose gel and DNA was extracted with QIAquick Gel Extraction Kit (Qiagen, Valencia, CA). The purified PCR fragments were cloned using CloneJET PCR cloning kit (Thermo Scientific, Pittsburgh, PA) according to the manufacturer’s instructions. An average of 20 clones per sample was sequenced at Eurofins Genomics (Huntsville, AL). The output was processed using Sequencher 5.2 (Gene Codes Corp., Ann Arbor, MI).

### Statistical analysis

Statistical analyses were conducted using SPSS v22.0 and R v3.1.2. As a quality control step, principle components analysis of methylation from the 450K array data using all three tissue sources was run to ensure that placental and blood samples were not cross-contaminated before proceeding with primary analyses. As the dependent variable, methylation, lay on a beta distribution of 0 (fully unmethylated) to 1 (fully methylated), beta regression was used to test associations of the individual CpG sites with chronic stress and war trauma using the betareg package for R [95]. Infant sex was included as a covariate in analyses of cord blood and placenta [96]. To address multiple testing, *q* values were computed to estimate false discovery rate (FDR) with *q* < .25 demonstrating “moderate confidence” of statistically significant findings [97].

We intentionally did not contrast methylation levels in this population to control groups generated from Western populations or other geographic areas as a non-stressed comparison group. Rather, a wide range of stressors was reported among the women in this study, with some reporting few stressors. This provided sufficient natural variation to examine the associations of maternal stress and methylation within a population of women controlling for geographic and cultural context.

Given the novelty of examining *BDNF* methylation in three tissues simultaneously, and the fact that *p* values and other statistics derived from them are in themselves arbitrary thresholds which may overlook many variants with smaller but real effects [98–100], a two-pronged approach was employed for identifying loci of interest for future research: (a) those that meet strict statistical significance criteria based on both *p* < .05 and *q* < .25, regardless of what, if anything, is known about those sites, and (b) those that meet the unadjusted *p* < .05 level of significance but are potentially important based on being located at putative transcription factor binding sites and thus of interest for future studies examining BDNF expression [45, 57] or prior evidence of association, as has been recommended for and used in genetic studies [46, 100–102].

## Additional file

Additional file 1: Table S1. Position of significant CpG sites in relation to putative transcription factors, according to source. **Table S﻿﻿2.** CpG sites identified via bisulfite sequencing with their genomic locations and positions.**Figure S1.** Genomic re﻿gion of Chr11:27722975-27723350 (GRCh37/hg19) representing the cloned sequence in placental tissue.

## Abbreviations

BDNF: Brain-derived neurotrophic factor
cAMP: Cyclic adenosine monophosphate
CRE: cAMP response element
FDR: False discovery rate
HPA: Hypothalamic-pituitary-adrenocortical
MeCP2: Methyl-CpG-binding protein 2
TFB: Transcription factor binding
