# Supplementary material for: BNDF methylation in mothers and newborns is associated with maternal exposure to war trauma
Source: Clin Epigenetics. 2017 Jun 30;9:68. doi: 10.1186/s13148-017-0367-x (PMC5493129; doi:10.1186/s13148-017-0367-x)
Supplement: Additional file 1: Table S1. — Position of significant CpG sites in relation to putative transcription factors, according to source. Table S﻿﻿2. CpG sites identified via bisulfite sequencing with their genomic locations and positions.Figure S1. Genomic re﻿gion of Chr11:27722975-27723350 (GRCh37/hg19) representing the cloned sequence in placental tissue. [file 13148_2017_367_MOESM1_ESM.docx]

**Supplemental Information**

**Supplementary Table 1 Position of significant CpG sites in relation to putative transcription factors, according to source**

| **Transcription factor or regulatory element** | **CpG site** | **Location (GRCh37/hg19)** | **ENCODE** | | **PhysBinder with average precision (Location: GRCh37/hg19)** | **Motifmap**  **(Location: GRCh37/hg19)** | **HMR Conservation**  **(Location: GRCh37/hg19)** |
| --- | --- | --- | --- | --- | --- | --- | --- |
|  |  |  | **Binding region** | **Peak position** |  |  |  |
| Ahr, Arnt, HIF-1 | cg12448003 | chr11:27742366-27742366 | - | - | - | chr11:27742362-27742371 | - |
|  | cg06684850 | chr11:2774369-27742369 | - | - | - | chr11:27742362-27742371 | - |
| BHLHB2 | cg12448003 | chr11:27742366-27742366 | - | - | chr11:27742359-27742367 | - | - |
| CTCF | cg09492354 | chr11:27720710-27720710 | chr11:27720628-27720740 | 27720691 | - | - | - |
|  | cg26840770 | chr11:27723291-27723291 | chr11:27723110-27723309 | 27723210 | - | - | - |
|  | cg26949694 | chr11:27742061-27742061 | chr11:27741948-27742111 | 27742030 | - | - | - |
|  | cg24249411 | chr11:27744760-27744760 | chr11:27744666-27744909 | 27744788 | - | - | - |
| Esrrb | cg27193031 | chr11:27721088-27721088 | - | - | chr11:27721080-27721089 | - | - |
|  | cg25962210 | chr11:27721223-27721223 | - | - | chr11:27721223-27721232 | - | - |
| GATA1 | cg16257091 | chr11:27743580-27743580 | - | - | chr11:27743573-27743580 | - | - |
| Myc | cg12448003 | chr11:27742366-27742366 | - | - | - | chr11:27742362-27742369 | - |
|  | cg06684850 | chr11:27742369-27742369 | - | - | - | chr11:27742362-27742369 | - |
| N-Myc | cg12448003 | chr11:27742366-27742366 | - | - | chr11:27742363-27742368 | chr11:27742359-27742371 | - |
|  | cg06684850 | chr11:27742369-27742369 | - | - | chr11:27742363-27742369 | chr11:27742359-27742371 | - |
| Pol2/Pol2-4H8 | cg09492354 | chr11:27720710-27720710 | chr11:27720367-27720776 | 27720650 | - | - | - |
|  | cg26840770 | chr11:27723291-27723291 | chr11:27722833-27723292 | 27723097 | - | - | - |
| POU3F2 | cg26840770 | chr11:27723291-27723291 | - | - | - | - | chr11:27723289-27723304 |
| SPI1 | cg01225698 | chr11:27742355-27742355 | - | - | chr11:27742345-27742356 | - | - |
| STAT5A | cg26840770 | chr11:27723291-27723291 | - | - | - | - | chr11:27723270-27723293 |
| Tfcp2l1 | cg27193031 | chr11:27721088-27721088 | - | - | chr11:27721082-27721091 | - | - |
|  | cg17413943 | chr11:27739827-27739827 | - | - | chr11:27739826-27739839 | - | - |
|  | cg26949694 | chr11:27742061-27742061 | - | - | chr11:27742052-27742061 | - | - |
|  | cg07704699 | chr11:27742833-27742833 | - | - | chr11:27742830-27742839 | - | - |
| USF1 | cg12448003 | chr11:27742366-27742366 | - | - | - | chr11:27742361-27742368 | - |
| USF2 | cg12448003 | chr11:27742366-27742366 | - | - | chr11:27742361-27742370 | chr11:27742362-27742368 | - |
|  | cg06684850 | chr11:27742369-27742369 | - | - | chr11:27742361-27742370 | - | - |

**Supplementary Table 2 CpG sites identified via bisulfite sequencing with their genomic locations and positions**

| **CpG** | **Location (GRCh37/hg19)** | **CpG position in the sequence** | **Percent methylated** |
| --- | --- | --- | --- |
| **CpG1** | chr11:27723049 | 75 | 0.00% |
| **CpG2** | chr11:27723062 | 88 | 0.00% |
| CpG3 | chr11:27723075 | 101 | 0.06% |
| CpG4 | chr11:27723095 | 121 | 0.06% |
| CpG5 | chr11:27723125 | 151 | 1.80% |
| CpG6 | chr11:27723128 | 154 | 1.80% |
| **CpG7** | chr11:27723137 | 163 | 0.00% |
| **CpG8** | chr11:27723143 | 169 | 0.00% |
| CpG9 | chr11:27723159 | 185 | 0.06% |
| **CpG10** | chr11:27723161 | 187 | 0.00% |
| **CpG11** | chr11:27723190 | 216 | 0.00% |
| CpG12 | chr11:27723203 | 229 | 1.80% |
| **CpG13** | chr11:27723214 | 240 | 0.00% |
| **CpG14** | chr11:27723218 | 244 | 0.00% |
| CpG15 | chr11:27723237 | 263 | 0.06% |
| CpG16 | chr11:27723245 | 271 | 1.20% |
| **CpG17** | chr11:27723266 | 292 | 0.00% |
| CpG18 | chr11:27723290 | 316 | 1.90% |
| **CpG19** | chr11:27723327 | 353 | 0.00% |

*Note.* CpG sites that were fully unmethylated in all placental samples are bolded.

**Supplementary Figure 1 Genomic region: Chr11:27722975-27723350 (GRCh37/hg19) representing the cloned sequence in placental tissue.**
